# A Web of Science-Based Bibliometric Analysis of Global Noma Publications

**DOI:** 10.3390/tropicalmed7080198

**Published:** 2022-08-21

**Authors:** Diego Azañedo, Fabriccio J. Visconti-Lopez, Akram Hernández-Vásquez

**Affiliations:** 1 Facultad de Ciencias de la Salud, Universidad Científica del Sur, Lima 15067, Peru; 2 Independent Researcher, Lima 15468, Peru; 3 Centro de Excelencia en Investigaciones Económicas y Sociales en Salud, Vicerrectorado de Investigación, Universidad San Ignacio de Loyola, Lima 15024, Peru

**Keywords:** noma, cancrum oris, neglected diseases, bibliometrics, trends

## Abstract

The World Health Organization recognizes noma as a global health problem and has suggested prioritizing research into this disease. A bibliographic search of original articles published in the Web of Science database up to 2022 was performed. A bibliometric analysis was carried out with the bibliometrix package in R and VOSviewer. We identified 251 articles published in 130 journals. The first publication was in 1975, the highest number of publications was in 2003, and the average number of citations per document was 12.59. The author with the highest number of publications was Enwonwu CO, and the Noma Children’s Hospital had the highest number of articles on this topic. *Plastic and Reconstructive Surgery* was the journal with the most publications, and the study by Petersen PE was the most cited. The country of corresponding authors that had the most publications and the most significant number of total citations was the United States. “Children” and “Reconstruction” were the most used keywords. In conclusion, there are few publications on noma worldwide, confirming the neglected status of this disease. Urgent actions are needed to increase evidence in regard to the epidemiology of noma and public health interventions to mitigate the ravages of this disease.

## 1. Introduction

Noma disease, also known as cancrum oris, is severe gangrene of the orofacial region that most commonly affects children [1]. This disease begins as a gingival lesion that quickly spreads through the mouth and throat tissues, leaving aesthetic and functional scars in the affected anatomical region [1]. Although the etiology is unknown, it is thought to be a combination of factors such as malnutrition, immunosuppression, and intraoral infection [2]. In 1998, the global annual incidence of noma was estimated at 140,000 cases, with a prevalence of 770,000 noma survivors, and a life expectancy of 40 years after diagnosis [3,4]. Although cases of noma have been reported in countries on all continents, most cases occur in sub-Saharan countries such as Niger, Senegal, Burkina Faso, and Nigeria, with an incidence estimated of 25,600 cases per year and a mortality rate of 8.5%, which can reach 85% without treatment [3,5,6]. In addition, it has been estimated that this disease generates a burden of approximately 1.1 million disability-adjusted life years (DALYs) globally [7].

Despite its high mortality and burden, noma is a disease that receives little attention from the scientific and health community worldwide [8]. On the one hand, the World Health Organization (WHO) does not recognize it as a neglected tropical disease (NTD) despite meeting the four necessary criteria (stigma and mortality in precarious areas; being from a tropical area such as Africa; control and eradication are feasible with known public health strategies; little research and little documentation) [9]. On the other hand, noma does not appear within the estimates of the DALYs of the Global Burden of Disease (GBD) of 2019, despite presenting a higher global burden of morbidity than other diseases cataloged by the WHO as NTDs [8]. All of this causes greater ignorance among public health decision-makers and health personnel, resulting in late diagnosis and treatment, leading to disease progression with sequela or death [9].

A bibliometric study allows quantitative analysis of published articles and their activity indicators with respect to the number of publications and their productivity over a given period of time [10]. This type of analysis is less susceptible to researcher bias compared to narrative studies, as it provides results of statistical and quantitative analysis [11].

The WHO recognizes noma as a global health problem and recommends prioritizing its detection, eradication, epidemiological surveillance, and research [12,13]. Knowing the state of research trends related to noma over the years will help to incentivize interest in this disease and guide future research. Therefore, this research aimed to determine the bibliometric characteristics of scientific articles, such as the number of articles and trends according to years, authors with the highest number of publications, institutions with the highest number of publications, publications with the highest number of citations, journals with the highest number of publications, number of publications according to the correspondent’s country, countries with the largest joint publications on the subject and an analysis of the characteristics of the keywords, on noma disease at a global level. The indicators of this type of analysis can obtain information on trends, collaborations, the countries in which research is frequently carried out, the connection among authors and thematic diagrams such as maps [14]. Therefore, through this analysis, we can study the knowledge and development of research on a given topic with empirical evidence, as in the case of noma disease.

## 2. Materials and Methods

### 2.1. Data Source and Search Strategy

The Web of Science (WOS) database was chosen as the bibliographic data source for the study. WOS was selected because it is one of the most used databases for bibliometric studies [15] and it has not been used in previous bibliometric studies on noma. Impact factor and journal quartile were obtained from Journal Citation Reports.

The search strategy used was based on a previous systematic review [16] and was as follows: TS = (Noma OR Nomas OR “Gangrenous Stomatitis” OR “Gangrenous Stomatitides” OR “Cancrum Oris” OR “fusospirochaetal gangrene” OR “Stomatitis gangrenosa” OR “necrotising ulcerative stomatitis”) NOT TS = (“non-orthogonal” OR “nonorthogonal” OR wireless OR network OR 5G OR communication OR nitrosodimethylamine OR “Northern Manhattan Study”). There were no restrictions on languages, countries or regions, affiliations, journals, research areas, or WOS categories. The search date was 10 July 2022.

### 2.2. Data Collection

**Figure 1:**
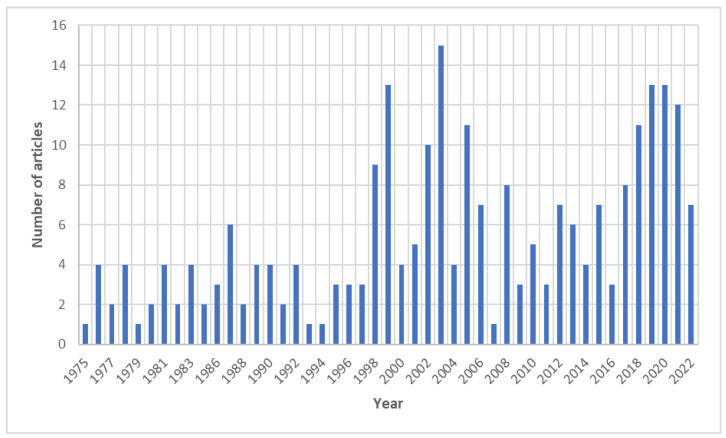
Evolution of the publication of articles on noma in the Web of Science from 1975 up to 10 July 2022.

The inclusion criteria were the following: (1) publications on noma; and (2) years of publication: up to 10 July 2022. A total of 753 records were retrieved and were manually reviewed by one author (FJVL) to verify that they met the inclusion criteria. All publications not related to noma were removed, and a total of 251 posts were ultimately included. The full records and cited references of all publications that met the inclusion criteria were exported and downloaded as .CIW files with “Full Record and Cited References” to standardize, analyze, and visualize the data. Manual standardization was performed to merge some authors, institutions, and keyword terms because different names, misspellings, or variations (e.g., plurals, synonyms) of words may occur in a thesaurus.txt file as described in the VOSviewer manual [17]. Figure 1 illustrates the search, selection, and analysis process.

### 2.3. Bibliometric Analysis

Bibliometric and network indicators were used to analyze the publications obtained. Quantitative bibliometric indicators such as the evolution of annual production, most productive authors, most productive countries, most cited articles, and journals with the highest number of publications were determined using the bibliometrix package in R (Version 4.0.2) [18]. VOSviewer (version 1.6.18) was used for the network analysis (co-authorship and co-occurrence of terms) of this study [19]. Author, organization, and country co-authorship maps, the journal co-citation map, and the keyword co-occurrence map (Keywords Plus) were constructed. The counting method used in this study was “Complete Count”. The normalization method applied was with “Association Strength”, and two standard weight attributes of “Documents” and “Occurrences” were applied. In network analysis, each of the nodes represents an element (author, organization, country, and keyword). The width of the connection line represents the strength of the link or co-occurrence, and the color indicates the average appearance of the year of origin [17].

### 2.4. Ethics

Ethical approval was not requested because this is a bibliometric study that does not involve humans or animals.

## 3. Results

A total of 251 papers on noma published in 130 sources were identified in the WOS database; the WOS codes of the included articles can be found in the supplementary material (Table S1). Of the articles included, 173 were original articles; 20 were editorials; 20 were letters; 19 were review articles; and 12 were conference abstracts. The first publication on the subject was in 1975, and the largest number of publications was in 2003 (15 articles) (Figure 1). An average of 12.6 citations per document was found.

**Figure 2:**
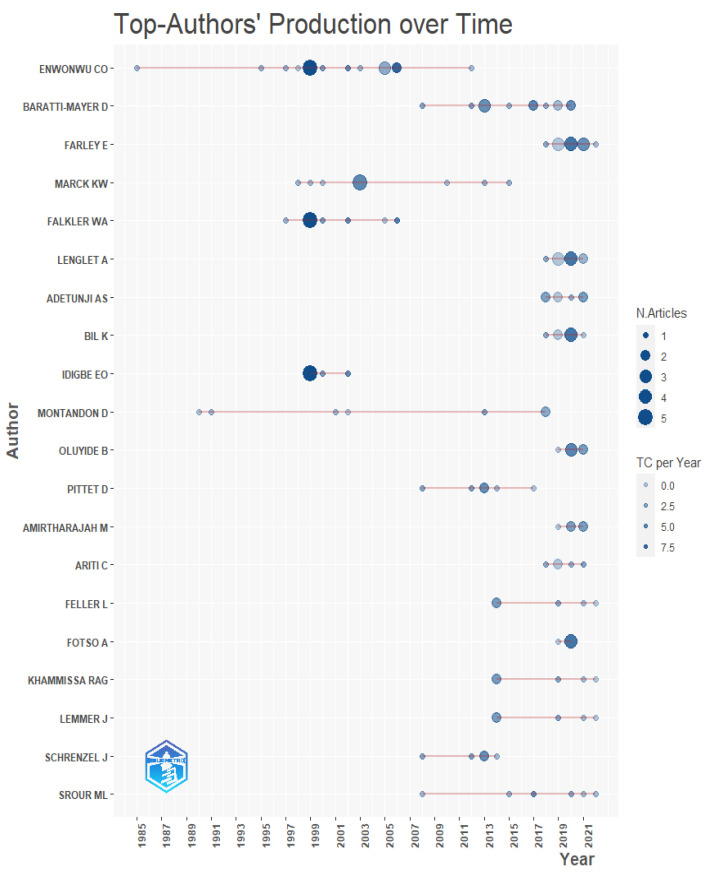
Scientific production of the top authors over time. TC: total citations.

**Figure 3:**
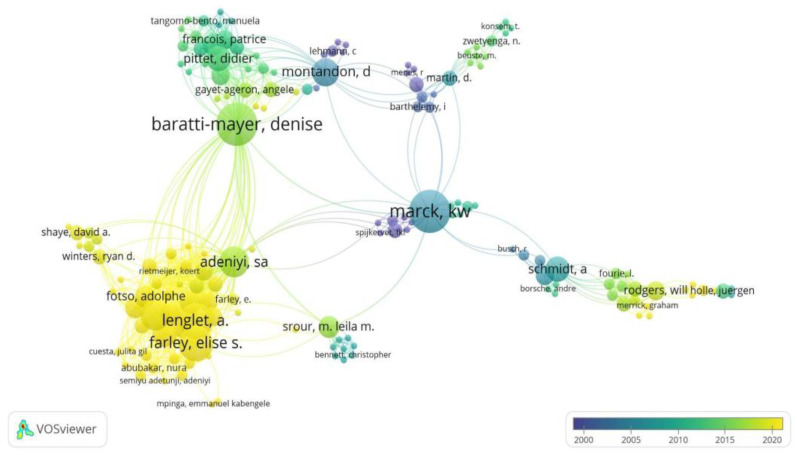
Network analysis of co-authorship.

In relation to the 20 authors with the highest scientific production on noma, the author with the most publications was Enwonwu CO, with 18 articles followed by Baratti-Mayer D (13 articles), for whom the majority of their work was published around 2015; and Farley E and Marck KW, both with 11 articles, who published the majority of their work around 2020 and 2005, respectively (Figure 2 and Figure 3).

The Petersen PE study, published in 2012, was the most cited, with a total of 396 citations and an average number of citations per year of 36.0. Likewise, this author published the most recently published article to enter the top 10 most cited articles. The studies by Paster BJ (2002) and Enwonwu CO (2006) occupied the second and third positions in terms of the number of citations, with 104 and 101 citations in total, with an average number of annual citations of 4.95 and 5.94, respectively (see Table 1).

The journals with the highest number of articles published were *Plastic and Reconstructive Surgery* (12 articles) and the *American Journal of Tropical Medicine and Hygiene* (11 articles), both of which are from the United States. Likewise, of the 10 journals with the highest number of publications, 5 belong to the United States, 4 to the United Kingdom and 1 to Germany (see Table 2).

**Figure 4:**
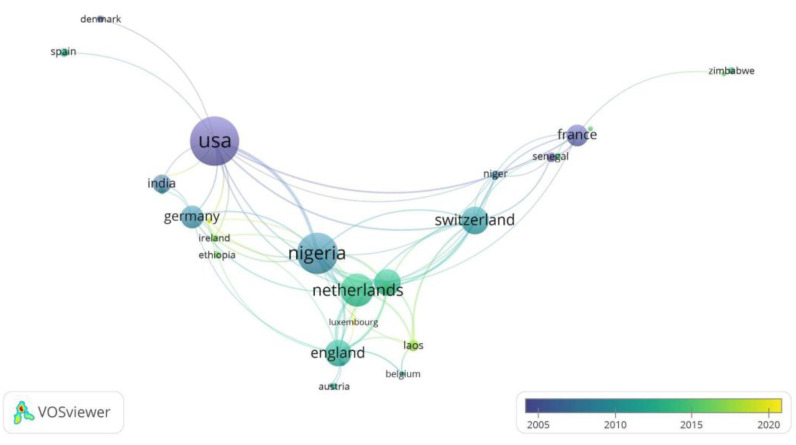
Network analysis of country co-authorship.

The countries of the corresponding authors with the highest number of articles on noma disease were the United States (42 articles), representing 19.2% of all articles, followed by Nigeria (18 articles) with 8.2% of the articles. Likewise, the highest percentage of single country publication (SCP) articles was held by Nigeria with 11/18 (61%), unlike Laos (0/5) with no SCP (see Table 3). Additionally, the highest number of total article citations was obtained by the United States (1017) and Switzerland (301) (see Table 3). The highest co-occurrence of countries was between the United States and Nigeria (Figure 4).

**Figure 5:**
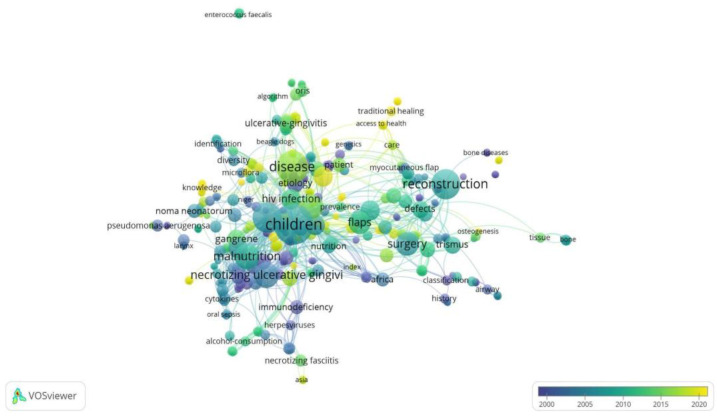
Network analysis of co-occurrence of terms.

**Figure 6:**
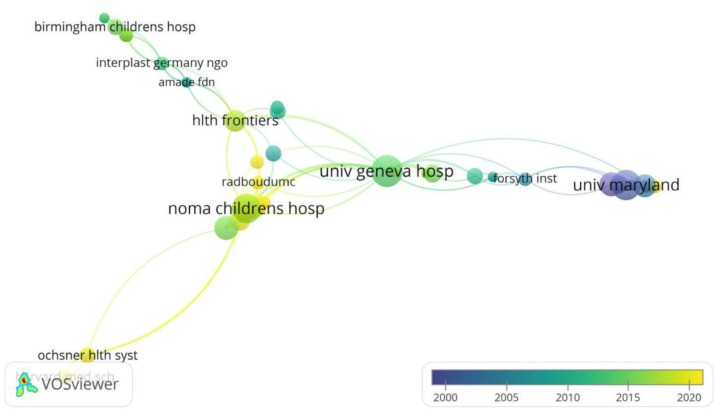
Network analysis of organization co-authorship.

Regarding keywords, the most used terms were “children” and “reconstruction” around 2005 and “disease” around 2015 (see Figure 5). Lastly, the institutional affiliations with the highest number of articles published on Noma disease were: Noma Children’s Hospital, University Geneva Hospital, and the University of Maryland (see Figure 6).

## 4. Discussion

The present study aimed to evaluate the bibliometric characteristics of the scientific production on noma disease in the WOS. In total, 251 documents published up to 10 July 2021 were identified, with the highest number of publications recorded in 2003 (15 articles). In general, the annual frequency of publications is variable and does not show a stable growth pattern. In the last full year evaluated (2021), only 12 publications on the subject were recorded. The low number of publications identified suggests little interest in the study of this disease on the part of the scientific community, which is negligent due to its impact on infant morbidity and mortality in neglected and extremely poor populations in the world [1].

The author with the highest number of publications on the subject was Enwonwu CO, with 18 publications. Likewise, this author published 5 of the 20 most cited articles identified in our study. The production of this author could be attributed to the fact of having been president of the Noma International Committee of the Noma Children’s Hospital, in Sokoto, Nigeria, which has been treating children with this condition for several years and has allowed access to closely study this disease [1,20,21]. According to the website of this institution, Dr. Enwonwu is currently affiliated with the University of Maryland in the United States [20]. Among the publications by this author is “Noma (Cancrum oris)”, a literature review on the disease published in the seminar section of the Lancet magazine in 2006 [1], which, according to our results, is also one of the most cited articles on the subject. “Oro-facial gangrene (Noma/cancrum oris): pathogenetic mechanisms” is another contribution by this author published in 2000, which aimed to synthesize knowledge about the pathogenesis of noma and present recommendations for the prevention of this disease [22]. To date, authors such as Baratti-Mayer D and Farley E occupy the second and third positions in the number of articles on noma. These authors usually publish on topics related to the epidemiology of noma, as well as opinion contributions in order to raise global awareness among stakeholders to include this disease as an NTD [8,9,23].

The most cited article on noma in the WOS is entitled “The global burden of periodontal disease: towards integration with chronic disease prevention and control”, by Petersen and Hiroshi, which addresses the issue of including oral diseases, mainly periodontal, within the WHO agenda for addressing chronic diseases [24]. Within the document, in the section “HIV/AIDS and periodontal health”, the authors mention noma as a periodontal onset disease, predominant in children from 1 to 4 years old and mainly associated with extreme poverty [24]. In relation to this, several authors have recommended the need to include noma as an NTD by the WHO because it meets the definition [9,25]. It is important to highlight that at the seventh plenary meeting of the 74th WHO General Assembly, one of the requests to the WHO Director General was to include noma in the revision process planned by the WHO in 2023 and to consider noma in the roadmap of NTDs between the years 2021 and 2030 [26,27]. This demonstrates the potential of scientific publications to influence the global public health agenda. The second most cited article on the subject belongs to Paster et al. and is called “Prevalent bacterial species and novel phylotypes in advanced Noma lesions”, in which molecular methods identified up to 67 bacterial species or phylotypes in DNA samples isolated from children with noma [28].

The journal with the highest number of publications on noma was *Plastic and Reconstructive Surgery*, from the United States, with an impact factor of 5.169 [29]. This journal aims to inform readers about developments in areas related to reconstructive and cosmetic surgery [30]. Noma is characterized as a disease that destroys the skin and underlying tissues of the face and the oral cavity, disfiguring the face, and thus, it is to be expected that some of the publications would be oriented to the reconstruction of the lesions caused by this disease [31]. The journals that occupy the second and fourth positions in terms of production on noma are the *American Journal of Tropical Medicine and Hygiene* and *PLOS Neglected Tropical Diseases*, which can be justified in that noma is considered a neglected tropical disease, and these high-impact journals have been used as a window to highlight this concern to the scientific community [3,9,32], mainly among those interested in research on this type of disease.

In relation to the countries with corresponding authors with the largest number of publications on noma, the United States and Nigeria are the main contributors in terms of production on this disease in the world, which also reflects the collaboration of these two countries in research on the topic. This pattern has been previously described in a bibliometric analysis of noma in Scopus [33]. In the first place, the United States is one of the countries with the highest spending per capita on research. Likewise, it is where the University of Maryland is located, which in the 2000s was one of the largest contributors to the production on noma, with the work led by Enwonwu CO. Nigeria, on the other hand, is one of the countries hardest hit by this disease, and it is where the Noma Children’s Hospital in Sokoto is located [34]. This institution is specialized in the management of children with noma and is where Enwonwu CO was director between 1999 and 2004, and where research on the disease continues to this day [20]. Similarly, the United States and Nigeria are the first and third countries with the highest number of citations in articles published on noma, followed by Switzerland, which also ranks fifth in the list of corresponding authors and is home to the University of Geneva Hospital, one of the institutions with the highest number of publications. In this regard, the University of Geneva houses the Institute of Global Health, which has an ongoing project that involves an international multidisciplinary consortium aimed at researching the causes of noma and its control since 2019 [35,36].

The keywords most frequently used by scientific articles published around 2005 were “children” and “reconstruction”, probably due to the greater number of published scientific articles that expose the high prevalence of the disease in children living in situations of extreme poverty. Likewise, a large part of these studies may be aimed at mitigating the facial disfigurement that occurs in affected infants in advanced stages of the disease, which could have social and psychological consequences for the infant. Finally, towards the year 2015, the word “disease” was one of the most frequently used in scientific articles, probably because opinion articles and comments regarding the inclusion of these diseases as NTDs were more frequently observed.

Among the limitations of this study, it should be mentioned that although some of the studies included in the analysis are mentioned, critical evaluation of their content and an analysis of their quality are beyond the objective of a bibliometric study. On the other hand, the use of only the WOS database may limit the scope of the publications on the subject, making it impossible to count the publications of other databases that index biomedical journals such as PubMed or Scopus or those in regional databases or gray literature. However, the WOS is one of the largest databases that includes the indexing of scientific publications from different subject areas, including biomedicine, and has tools that are ideal for downloading metadata for analysis [15].

## 5. Conclusions

The low number of publications on noma in the WOS reflects that it is a neglected disease, which has been previously reported by another recent bibliometric research study using the Scopus database. Urgent action is required by government bodies and agencies to promote and finance the generation of new research to improve the understanding of the etiological and contributing factors of this disease. Likewise, part of the research should evaluate the impact of the implementation of preventive programs at different levels in the population at risk and with the disease, as well as the involvement of the health personnel in charge of their care. Only in this way will it be possible to mitigate the social and health impact of this disease in line with the WHO agenda to consider noma in the roadmap of NTDs between the years 2021 and 2030.

## Figures and Tables

**Table 1:** Top 20 most cited articles on noma.

N	Article	DOI	TC	TC Per Year	NTC
1	Petersen PE, 2012	10.1111/j.1600-0757.2011.00425.x	396	36.0	5.9
2	Paster BJ, 2002	10.1128/JCM.40.6.2187-2191.2002	104	5.0	5.8
3	Enwonwu CO, 2006	10.1016/S0140-6736(06)69004-1	101	5.9	4.1
4	Horning GM, 1995	10.1902/jop.1995.66.11.990	84	3.0	1.7
5	Enwonwu CO, 2000	10.1177/10454411000110020201	79	3.4	3.2
6	Enwonwu CO, 1999	10.4269/ajtmh.1999.60.223	78	3.3	2.9
7	Falkler WA, 1999	NA	58	2.4	2.2
8	Falkler WA, 1999	10.4269/ajtmh.1999.60.150	48	2.0	1.8
9	Slots J, 2003	10.1111/j.1875-595X.2003.tb00771.x	47	2.4	3.1
10	Enwonwu CO, 1995	NA	47	1.7	1.0
11	Adekeye EO, 1983	10.1016/S0301-0503(83)80040-X	47	1.2	3.1
12	Marck KW, 2003	10.1097/01.PRS.0000055445.84307.3C	45	2.3	2.9
13	Enwonwu CO, 1985	10.1111/j.1600-0528.1985.tb00443.x	43	1.1	1.7
14	Fieger A, 2003	10.1046/j.1365-3156.2003.01036.x	42	2.1	2.7
15	Bourgeois DM, 1999	NA	42	1.8	1.6
16	Barmes DE, 1997	10.1046/j.1365-3156.1997.d01-220.x	42	1.6	1.9
17	Huyghe A, 2008	10.1128/AEM.01722-07	41	2.7	3.0
18	Montandon D, 1991	10.1097/00006534-199101000-00013	41	1.3	1.7
19	Chidzonga MM, 1996	10.1016/S0278-2391(96)90159-7	37	1.4	1.6
20	Herrera D, 2018	10.1111/jcpe.12941	36	7.2	5.7

NA: not available. TC: total citations. NTC: normalized total citation.

**Table 2:** Top 10 journals with the highest number of publications.

N	Journal	Freq	IF	Quartile
1	*Plastic and Reconstructive Surgery*	12	5.169	Q1
2	*American Journal of Tropical Medicine and Hygiene*	11	3.707	Q2
3	*Oral Diseases*	10	4.068	Q1
4	*PLOS Neglected Tropical Diseases*	10	4.781	Q1
5	*British Journal of Oral & Maxillofacial Surgery*	7	2.108	Q3
6	*British Journal of Plastic Surgery*	6	1.291	Q2
7	*Journal of Craniofacial Surgery*	6	1.172	Q4
8	*Lancet*	6	202.731	Q1
9	*European Journal of Plastic Surgery*	5	NA	NA
10	*Journal Of Dental Research*	5	8.924	Q1

IF: impact factor. NA: not available. Q: quartile. Source of IF and quartile: Journal Citation Reports™ 2021.

**Table 3:** Top 10 countries of authors with publications on noma.

N	Article	Articles	%	SCP	MCP	Total Citations
1	United States	42	19.2	29	13	1017
2	Nigeria	18	8.2	11	7	238
3	Netherlands	17	7.8	11	6	220
4	United Kingdom	17	7.8	12	5	105
5	Switzerland	15	6.8	10	5	301
6	France	12	5.5	9	3	47
7	Germany	12	5.5	11	1	154
8	India	12	5.5	11	1	62
9	South Africa	9	4.1	9	0	67
10	Italy	5	2.3	5	0	24

SCP: single country publications. MCP: multiple country publications.

## Data Availability

Not applicable.

## Supplementary Materials

The following supporting information can be downloaded at: https://www.mdpi.com/article/10.3390/tropicalmed7080198/s1, Table S1: List of Accession Number of documents included in the study.
