# Supplementary material for: A Web of Science-Based Bibliometric Analysis of Global Noma Publications
Source: Trop Med Infect Dis. 2022 Aug 21;7(8):198. doi: 10.3390/tropicalmed7080198 (PMC9412599; doi:10.3390/tropicalmed7080198)
Supplement: Supplementary file 1 [file tropicalmed-07-00198-s001.zip › tropicalmed-1879859-supplementary.pdf]

Supplementary Materials

Table S1. List of Accession Number of documents included in the study

Accession Number	Results
UT=(WOS:000518550000005 OR WOS:000285547000004 OR WOS:000181962300018 OR WOS:000419724800021 OR WOS:A1977DL76000014 OR WOS:000176713600005 OR WOS:000335147300035 OR WOS:000290768600005 OR WOS:000408749700007 OR WOS:000435795300007 OR WOS:A1987H257900009 OR WOS:000713327100552 OR WOS:000326512300032 OR WOS:000577093000022 OR WOS:000182653300005 OR WOS:000259418200001 OR WOS:000438672900013 OR WOS:000787579000001 OR WOS:000519558600003 OR WOS:000291566000003 OR WOS:A1976BH99200011 OR WOS:000354313200020 OR WOS:000226808201768 OR WOS:000227610705055 OR WOS:A1981MB57100005 OR WOS:000176024702898 OR WOS:000302132100013 OR WOS:000221561500020 OR WOS:A1980LQ55800006 OR WOS:A1979HY80100005 OR WOS:000072574700023 OR WOS:A1992HR71500002 OR WOS:A1983RE80700003 OR WOS:A1981LJ98800011 OR WOS:000073852700010 OR WOS:000072606400011 OR WOS:000430432800033 OR WOS:000185279700013 OR WOS:000220769900003 OR WOS:000291120900040 OR WOS:000168379500013 OR WOS:A1989AV80300011 OR WOS:A1995RG53600030 OR WOS:A1991EU60600015 OR WOS:A1992HX12500023 OR WOS:000179138700007 OR WOS:000075130100009 OR WOS:000504321700102 OR WOS:A1992KC20600015 OR WOS:000679975200015 OR WOS:000580818500067 OR WOS:A1989AR98100006 OR WOS:A1990CX07200006 OR WOS:000168481800014 OR WOS:000087689600004 OR WOS:A1988P308500049 OR WOS:A1982PQ26800010 OR WOS:A1981LC37200370 OR WOS:000529353700086 OR WOS:000284463500039 OR WOS:000169377000001 OR WOS:000180119600006 OR WOS:000085169200013 OR WOS:000478662500055 OR WOS:A1976BM46400003 OR WOS:000439165500014 OR WOS:000404962700085 OR WOS:A1978FK59300004 OR WOS:000580782700025 OR WOS:000385914300026 OR WOS:A1990DV59100027 OR WOS:000301776000002 OR WOS:000223926800013 OR WOS:000732467500005 OR WOS:A1989CD35200048 OR WOS:A1989AE42700045 OR WOS:A1986F910700009 OR WOS:000336422400016 OR WOS:A1983QU84400019 OR WOS:000314386600004 OR WOS:000530707700021 OR WOS:A1985ALM7000021 OR WOS:000073335400006 OR WOS:000796085200001 OR WOS:000173306400024 OR WOS:000171226900175 OR WOS:000078436300027 OR WOS:000697588600008 OR WOS:000548903800035 OR WOS:000797995100044 OR WOS:000263226700024 OR WOS:000083749400013 OR WOS:000270369100057 OR WOS:000283761500007 OR WOS:000324920800052 OR WOS:000083749400012 OR WOS:000230860100004 OR WOS:000367377400020 OR WOS:000220769900001 OR WOS:000254943300001 OR WOS:000675323800001 OR WOS:000316750100022 OR WOS:000075938800017 OR WOS:000690961900024 OR WOS:000234750200009 OR WOS:000384938200013 OR WOS:000173121900025 OR WOS:000236242400011 OR WOS:000184766700003 OR WOS:A1990EM12800009 OR WOS:A1995TF54900010 OR WOS:A1993MG02000001 OR WOS:000182449100021 OR WOS:000220769900004 OR WOS:000240698700022 OR WOS:000360939500012 OR WOS:000188766100016 OR WOS:A1987H747600009 OR WOS:A1983RF15600012 OR WOS:000240698700021 OR WOS:000798512700001 OR WOS:A1995RV52900017 OR WOS:A1983RV46200010 OR WOS:000234661100001 OR WOS:000238917200029 OR WOS:A1996VF41000012 OR WOS:000182834500008 OR WOS:A1996VG41100004 OR WOS:000253648400009 OR WOS:000329291500001 OR WOS:000730050800002 OR WOS:000467953000012 OR WOS:000083749400011 OR WOS:000230992700027 OR WOS:000346701000001 OR WOS:A1976BJ19500003 OR WOS:000332095600005 OR WOS:000613137500004 OR WOS:000427370200004 OR WOS:000765763900001 OR WOS:000471892500002 OR	251

WOS:A1985APU9200008 OR WOS:000176793000026 OR WOS:A1981LX80200003 OR WOS:000401896100042 OR WOS:000303901400025 OR WOS:000416117700017 OR WOS:A1987K745900018 OR WOS:000257118700004 OR WOS:A1986E328600006 OR WOS:000182156800011 OR WOS:000254943300004 OR WOS:A1982PN25900013 OR WOS:A1987K119600017 OR WOS:A1977EC90200008 OR WOS:A1992JT64900020 OR WOS:A1978FK25900006 OR WOS:000528508000020 OR WOS:000218920300001 OR WOS:000469895500013 OR WOS:000229588300018 OR WOS:000493064400171 OR WOS:000810670200001 OR WOS:000454949300029 OR WOS:000493390900008 OR WOS:000186973400014 OR WOS:A1976BC81700005 OR WOS:000670710200004 OR WOS:000804767400001 OR WOS:000336422400003 OR WOS:000710285300008 OR WOS:000484232000040 OR WOS:A1996WA52600017 OR WOS:000230992700009 OR WOS:000185144400001 OR WOS:000185144400002 OR WOS:000355670600002 OR WOS:000401767400004 OR WOS:000083749400014 OR WOS:000083892600012 OR WOS:000220769900002 OR WOS:000075399000002 OR WOS:000079990800024 OR WOS:000075399000003 OR WOS:000401767400002 OR WOS:000368501500001 OR WOS:000416392700013 OR WOS:000254065600024 OR WOS:A1997WE35300006 OR WOS:000636460300012 OR WOS:A1987G148500685 OR WOS:A1980JF69100012 OR WOS:000087783900002 OR WOS:000593282100004 OR WOS:000078878700010 OR WOS:A1978GJ18800007 OR WOS:000458122600004 OR WOS:000355372200013 OR WOS:000408394700004 OR WOS:000174211900019 OR WOS:000174211900020 OR WOS:000261323900022 OR WOS:000305514700011 OR WOS:000176159200047 OR WOS:000228286900009 OR WOS:000078383000002 OR WOS:A1975AB59700018 OR WOS:000519535600011 OR WOS:A1978FT51300011 OR WOS:000077149900018 OR WOS:000256513300011 OR WOS:000266674600032 OR WOS:000258012700025 OR WOS:000073015400025 OR WOS:000520180100138 OR WOS:000222339600010 OR WOS:A1994QH76000018 OR WOS:000475550400009 OR WOS:000273078600057 OR WOS:000493064400563 OR WOS:000443381000018 OR WOS:000336422400018 OR WOS:000169377000003 OR WOS:000507364502495 OR WOS:000502057400284 OR WOS:A1988Q660100011 OR WOS:000232659600019 OR WOS:000083749400015 OR WOS:000226928200009 OR WOS:000084946500001 OR WOS:000308092400002 OR WOS:000083749400001 OR WOS:000071553200001 OR WOS:A1990CL95600006 OR WOS:000308092400005 OR WOS:000087447100009 OR WOS:000538145000016 OR WOS:000434303800049 OR WOS:000228884600016 OR WOS:000452567400005 OR WOS:A1991EQ14300013 OR WOS:000237215700004 OR WOS:000239048700050 OR WOS:A1986A967800006 OR WOS:000083749400016 OR WOS:000366811400022 OR WOS:000274996000015 OR WOS:A1987L018000017 OR WOS:000227990500005 OR WOS:000692198200013 OR WOS:000564075100007)	
